# Substance use disorders, psychiatric disorders, and mortality after release from prison: a nationwide longitudinal cohort study

**DOI:** 10.1016/S2215-0366(15)00088-7

**Published:** 2015-04-28

**Authors:** Zheng Chang, Paul Lichtenstein, Henrik Larsson, Seena Fazel

**Affiliations:** a Department of Psychiatry, Warneford Hospital, University of Oxford, Oxford, UK; b Department of Medical Epidemiology and Biostatistics, Karolinska Institutet, Stockholm, Sweden

## Abstract

**Background:**

High mortality rates have been reported in people released from prison compared with the general population. However, few studies have investigated potential risk factors associated with these high rates, especially psychiatric determinants. We aimed to investigate the association between psychiatric disorders and mortality in people released from prison in Sweden.

**Methods:**

We studied all people who were imprisoned since Jan 1, 2000, and released before Dec 31, 2009, in Sweden for risks of all-cause and external-cause (accidents, suicide, homicide) mortality after prison release. We obtained data for substance use disorders and other psychiatric disorders, and criminological and sociodemographic factors from population-based registers. We calculated hazard ratios (HRs) by Cox regression, and then used them to calculate population attributable fractions for post-release mortality. To control for potential familial confounding, we compared individuals in the study with siblings who were also released from prison, but without psychiatric disorders. We tested whether any independent risk factors improved the prediction of mortality beyond age, sex, and criminal history.

**Findings:**

We identified 47 326 individuals who were imprisoned. During a median follow-up time of 5·1 years (IQR 2·6–7·5), we recorded 2874 (6%) deaths after release from prison. The overall all-cause mortality rate was 1205 deaths per 100 000 person-years. Substance use disorders significantly increased the rate of all-cause mortality (alcohol use: adjusted HR 1·62, 95% CI 1·48–1·77; drug use: 1·67, 1·53–1·83), and the association was independent of sociodemographic, criminological, and familial factors. We identified no strong evidence that other psychiatric disorders increased mortality after we controlled for potential confounders. In people released from prison, 925 (34%) of all-cause deaths in men and 85 (50%) in women were potentially attributable to substance use disorders. Substance use disorders were also an independent determinant of external-cause mortality, with population attributable fraction estimates at 42% in men and 70% in women. Substance use disorders significantly improved the prediction of external-cause mortality, in addition to sociodemographic and criminological factors.

**Interpretation:**

Interventions to address substance use disorders could substantially decrease the burden of excess mortality in people released from prison, but might need to be provided beyond the immediate period after release.

**Funding:**

Wellcome Trust, Swedish Research Council, and the Swedish Research Council for Health, Working Life and Welfare.

## Introduction

Every year more than 30 million people circulate through prisons,^1^ and most will eventually return to their communities. The period after release is associated with high risk for various health outcomes,^2^ and many studies have provided an epidemiological description of high mortality in people released from prison compared with the general population.^3^, ^4^, ^5^, ^6^, ^7^, ^8^, ^9^, ^10^ In the USA, the ex-prisoner population was estimated as 5·4 million people,^11^ who account for about 12% of roughly 250 000 deaths from external causes every year.^12^ However, few studies have examined potential risk factors for these high mortality rates,^13^ and therefore, information for evidence-based prevention is scarce.^14^

Substance use disorders and psychiatric disorders are important potential risk factors that are highly prevalent in prisoners and potentially treatable.^15^, ^16^ Although a few studies of post-release mortality have reported data for these disorders,^3^, ^17^ none has fully examined the association between them and differentiated the contribution of individual diagnoses. Mortality from drug overdose has been reported to be particularly high after release from prison.^18^ However, whether substance use disorders and other psychiatric disorders are causally associated with post-release mortality is uncertain, because prisoners are usually from deprived socioeconomic backgrounds,^4^ and both criminal behaviour and most psychiatric disorders tend to cluster in families. Whether substance use and other psychiatric disorders can be used to predict post-release mortality is also unknown.

To inform preventive interventions for post-release mortality, additional research is needed to identify potentially modifiable risk factors. In this population-based longitudinal study of people released from prison, we investigated the association between psychiatric disorders and post-release mortality. We aimed to address three main questions: first, whether substance use disorders and other psychiatric disorders are associated with mortality after prison release; second, what are the population effects of identified risk factors on all-cause and external-cause mortality in people released from prison; and third, whether any of the identified risk factors can be used to predict external-cause mortality after release from prison.

**Research in context**

**Evidence before this study**

We searched PubMed from Jan 1, 2000, to Dec 31, 2014, for research about the association between psychiatric disorders and mortality in people released from prison. We used the following search terms: “psychiatric disorder*” or “mental disorder*” or “mental illness*” or “substance*”, and “death*” or “mortal*” or “suicid*” or “overdose*”, and “releas*”or “former*” or “ex”, and “prison*” or “incarce*” or “felon*” or “jail*” or “custod*” or “offend*”. We identified two systematic reviews of mortality in people released from prison, which identified 18 studies and 25 studies, respectively, up to January, 2011. Since Jan 1, 2011, we identified a further six studies. Although the results of these studies have consistently shown that people released from prison are at increased risk for death following release, none of these has fully examined the association between psychiatric disorders and post-release mortality. In 2012, Zlodre and Fazel called for further work to examine the contribution of mental illness on post-release mortality. Furthermore, we identified no studies that investigated the effects of familial (genetic and environmental) factors on the potential link between psychiatric disorders and post-release mortality.

**Implications of all the available evidence**

Alcohol and drug use disorders, identified before prison release, were associated with substantially increased all-cause mortality and external-cause mortality. Additionally, little evidence suggested that other psychiatric disorders diagnosed before prison release increased all-cause or external-cause mortality rates. Substance use disorders significantly improved the prediction of external-cause mortality when added to a model with known sociodemographic and criminological factors.

**Added value of this study**

Our study is the first to investigate the effect of substance use disorders and other psychiatric disorders on mortality after prison release, while taking into account both measured (sociodemographic and criminological) and unmeasured (familial) confounding factors. We also investigated individual causes of death, and noted that non-traffic accidents and suicide contributed to a large proportion of external-cause deaths. The use of a total population cohort of 47 326 individuals released from prison between 2000 and 2010 enabled us to provide precise effect sizes, and to estimate the possible population effect of substance use disorders on post-release mortality.

## Methods

### Study population

We used data from several population-based registers in Sweden, which were linked with unique personal identification numbers.^19^ We selected a cohort of all people who have been imprisoned in Sweden since Jan 1, 2000, and released before Dec 31, 2009. We followed up the study individuals from the day of release until death, emigration from Sweden, or the end of the study (Dec 31, 2009). We obtained records of all criminal convictions since 1973 from the National Crime Register. From the National Patient Register, we obtained diagnoses for all inpatient psychiatric hospital admissions in Sweden since 1973, and outpatient care since 2001. We used the Cause of Death Register to obtain data for dates and causes of all deaths since 1958. We collected data for yearly assessments of income, marital status, employment status, and education for all individuals aged 15 years or older using the Longitudinal Integration Database for Health Insurance and Labour Market Studies. We also identified the siblings of people in prison using the Multi-Generation Register. The study was approved by the Regional Ethics Committee at Karolinska Institutet, Stockholm, Sweden. All data were anonymised before they were analysed.

### Procedures

We defined psychiatric disorders as any psychiatric diagnoses before release from prison, using data from the National Patient Register, which were based on ICD-8 (1973–86, codes 290–315), ICD-9 (1987–96, codes 290–319), and ICD-10 (1997–2009, codes F00–F99). To investigate the differences between individual psychiatric disorders, the following disorders were investigated: alcohol use disorder (ICD-8: 291, 303; ICD-9: 291, 303, 305A; ICD-10: F10); drug use disorder (ICD-8: 304; ICD-9: 292, 304, 305 [except 305.A]; ICD-10: F11–F19); personality disorder (ICD-8: 301 [except 301.1]; ICD-9: 301 [except 301.B]; ICD-10: F60–61); attention deficit hyperactivity disorder (ADHD; ICD-8: not applicable; ICD-9: 314; ICD-10: F90); other developmental or childhood disorders (ICD-8: 308; ICD-9: 299A, 312, 313, 315; ICD-10: F80–F98 [except F90]). We assigned a hierarchical approach to differentiate between schizophrenia-spectrum disorder, bipolar disorder, depression, and anxiety disorder, categorised as follows: schizophrenia-spectrum disorder (ICD-8: 295, 297, 298.1–9, 299; ICD-9: 295, 297, 298 [except 298.A], 299; ICD-10: F20–F29), including schizoaffective disorders and delusional disorders; bipolar disorder (ICD-8: 296.1, 296.3, 296.8; 296A, 296C–296E, 296W; ICD-10: F30–31), but not schizophrenia-spectrum disorder; depression (ICD-8: 296.2, 296.9, 298.0, 300.4; ICD-9: 296B, 296X, 298A, 300E, 311; ICD-10: F32–39), but without schizophrenia-spectrum disorder or bipolar disorder; and anxiety disorder (ICD-8: 300 [except 300.4], 305, 307; ICD-9: 300 [except 300.E], 306, 308, 309; ICD-10: F40–48), but without schizophrenia-spectrum disorder, bipolar disorder, or depression. The use of National Patient Register for psychiatric research is well established, and the National Patient Register data have good-to-excellent validity for a range of psychiatric disorders.^20^, ^21^, ^22^, ^23^ We defined substance use disorder as any diagnosis of alcohol or drug use disorders.

The main outcome was death after release from prison. The Cause of Death Register includes all people who, at the time of death, were registered as residents in Sweden, regardless of whether death occurred in Sweden or abroad, and it covers more than 99% of all deaths in Swedish residents.^24^ The underlying and contributing (secondary) causes of death are coded according to ICD-10,^25^ based on death certificates issued by physicians or forensic doctors. We extracted both all-cause mortality data and mortality data separated by ICD chapter in accordance with the underlying cause of death. Within external-cause mortality (ie, deaths caused by environmental events and circumstances, ICD-10 Chapter XX),^25^ we further examined deaths by traffic and non-traffic accidents, suicide, and homicide. In keeping with previous work, we included undetermined deaths (ICD-10: Y10–Y34) as suicides.

We measured covariates, which were sex, age, immigration status (defined as being born outside of Sweden), criminological factors (length of incarceration [categorised into four levels], violent index offence, any previous violent crime), and sociodemographic factors (civil status [categorised into four levels], employment, and highest level of completed education [categorised into three levels], disposable income, and neighbourhood deprivation) at the year of release. We did not replace missing data by imputation or other methods in the principal analyses, but did so in a sensitivity analysis.

### Statistical analysis

We calculated mortality (the number of deaths divided by person-years at risk) and 95% CIs for all-cause death, 11 causes of death by ICD chapter, and subcauses of external causes. We used SAS 9.4 software for all statistical analysis.

We used Kaplan-Meier survival curves to show the timing of post-release mortality for prisoners with and without substance use disorders, and prisoners with and without any other psychiatric disorders. To explore the association between individual disorders and post-release mortality, we constructed Cox proportional hazard models for each diagnosis investigated. We calculated hazard ratios (HRs) with progressive adjustment for age and immigration status, criminological, and sociodemographic factors, sex, and alcohol and drug use disorders. We verified the proportional hazards assumption by visually inspecting the Kaplan-Meier Curves and testing with Schoenfeld residuals. To examine whether the association between psychiatric disorders and death was explained by familial factors, we used a sibling-comparison approach, which we did by fitting a fixed-effect model^26^ (stratified Cox regression) to the subsample of full-siblings who were imprisoned, to adjust for all unmeasured genetic and environmental factors that were shared by siblings.

In addition to being significantly associated with the outcome, a clinically important risk factor should also have substantial population effect or predictive validity to identify high-risk individuals. To assess the effect of psychiatric disorders on post-release mortality, we used the population attributable fraction (PAF). The PAF measures the proportion of deaths that can be attributed to a risk factor, with the assumption that a causal association exists. In the presence of confounding, PAF can be calculated as,^27^

$$\mathrm{Pr}(X=1|\text{Y}=1)(1-HR_{\text{a}}^{-1})$$

where Pr (X = 1 | Y = 1) is the probability of exposure given outcome and *HR_a_* is the adjusted HR. We calculated the CIs of PAF using the Bonferroni inequality method.^28^

To assess the incremental predictive validity of psychiatric disorders for post-release mortality, we examined whether substance use disorder or any other psychiatric disorder could improve the prediction of external-cause mortality in released prisoners. We focused on external-cause mortality because these deaths are potentially preventable, whereas other causes of death might be mainly caused by age. A good predictor should improve discrimination, calibration, and reclassification. We assessed discrimination—the ability of a model to distinguish between those who will and will not have the outcome—using Harrell's c-index, which varies from 0·5 to 1·0.^29^ The c-index has a similar interpretation to the area under the receiver operating curve, but is more appropriate for survival data characterised by censored observations. We statistically assessed calibration, which examines goodness of fit, using the likelihood ratio test. The likelihood ratio test suggests whether the addition of a new variable significantly improves the fit of a model. We assessed reclassification using change in Royston's *R^2^*, which shows whether the addition of a new variable improves the percentage of variance explained by the model.^30^ For incremental predictive validity analysis, we included, age, sex, immigration status, criminological factors, sociodemographic factors, and previous self-harm in the baseline model as a-priori factors.^17^ We followed STROBE guidelines (appendix).

### Role of the funding source

The funder of the study had no role in study design, data collection, data analysis, data interpretation, or writing of the report. ZC had full access to all the data in the study and ZC and SF had final responsibility for the decision to submit for publication.

## Results

We identified 47 326 people in prison during the study period, and followed them up for a total of 238 457 person-years after release. Table 1 shows baseline sociodemographic and criminological information and psychiatric diagnoses in men and women who were imprisoned. 18 563 (42%) of 43 840 men and 2233 (64%) of 3486 women had any psychiatric disorder, and prevalence of alcohol and drug use was high. We identified 1458 pairs of siblings who were both imprisoned.

**Table 1:** Baseline sociodemographic characteristics and follow-up data

		**Men (n=43 840)**	**Women (n=3486)**
Person-years at risk		221 522	16 935
Deaths during follow-up		2703 (6%)	171 (5%)
Age group (years)			
	16–24	8466 (19%)	361 (10%)
	25–39	17 291 (39%)	1409 (40%)
	≥40	18 083 (41%)	1716 (49%)
Civil status*			
	Unmarried	26 910 (65%)	1614 (49%)
	Married	5066 (12%)	537 (16%)
	Divorced	9105 (22%)	1094 (33%)
	Widowed	222 (<1%)	67 (2%)
Highest education (years)*			
	<9	19 546 (47%)	1765 (53%)
	9–11	19 174 (46%)	1322 (40%)
	≥12	2583 (6%)	225 (7%)
Employed*		8045 (20%)	355 (11%)
Immigrant		13 710 (31%)	806 (23%)
Disposable income*†		775 (473 to 1100)	749 (444 to 1082)
Neighbourhood deprivation*‡		0·38 (−0·17 to 1·48)	0·35 (−0·15 to 1·46)
Length of incarceration (months)			
	<6	30 155 (69%)	2608 (75%)
	6–11	7270 (16%)	506 (14%)
	12–23	4408 (10%)	283 (8%)
	≥24	2007 (5%)	89 (3%)
Violent index offence		17 294 (39%)	643 (18%)
Previous violent crime		23 960 (55%)	1112 (32%)
Previous psychiatric disorder			
	Any psychiatric disorder	18 563 (42%)	2233 (64%)
	Alcohol use disorder	9276 (21%)	968 (28%)
	Drug use disorder	9597 (22%)	1438 (41%)
	Personality disorder	2320 (5%)	353 (10%)
	Attention deficit hyperactivity disorder	546 (1%)	51 (1%)
	Other developmental or childhood disorder	979 (2%)	139 (4%)
	Schizophrenia-spectrum disorder	1237 (3%)	130 (4%)
	Bipolar disorder	216 (<1%)	35 (1%)
	Depression	2553 (6%)	418 (12%)
	Anxiety disorder	3247 (7%)	534 (15%)

Data are number (%) or median (IQR).

* 2573 men and 174 women had missing values for civil status, highest education, employment, disposable income, and neighbourhood deprivation.

† 100 Swedish Krona.

‡ Standardised score of the overall degree of socioeconomic deprivation in an individual's residential area.

During a median follow-up time of 5·1 years (IQR 2·6–7·5), 2874 (6%) of study individuals died after release from prison (table 2). Median age at death was 49·1 years (IQR 38·4–58·0). The median time from release to death was 3·5 years (1·7–5·4). 417 people (15% of deaths) died in the first year and 856 (30%) died within 2 years. The overall all-cause mortality rate was 1205 deaths per 100 000 person-years (table 2), and the rate was slightly higher in men than in women. 1276 deaths (44% of deaths) were attributed to external causes, which were mainly non-traffic accidents and suicides (table 2). Cardiovascular causes and cancer were other major causes of deaths.

**Table 2:** Mortality rates

		**Overall**		**Men**		**Women**	
		Deaths	Mortality	Deaths	Mortality	Deaths	Mortality
All causes		2874 (100%)	1205 (1161–1249)	2703 (100%)	1220 (1174–1266)	171 (100%)	1010 (858–1161)
Certain infectious and parasitic diseases (chapter I)		92 (3%)	39 (31–46)	82 (3%)	37 (29–45)	10 (6%)	59 (22–96)
Neoplasms (chapter II)		305 (11%)	128 (114–142)	282 (10%)	127 (112–142)	23 (13%)	136 (80–191)
Endocrine, nutritional and metabolic diseases (chapter IV)		39 (1%)	16 (11–21)	37 (1%)	17 (11–22)	2 (1%)	12 (0–28)
Mental and behavioural disorders (chapter V)		195 (7%)	82 (70–93)	185 (7%)	84 (71–96)	10 (6%)	59 (22–96)
Diseases of the nervous system (chapter VI)		27 (1%)	11 (7–16)	25 (1%)	11 (7–16)	2 (1·2%)	12 (0–28)
Diseases of the circulatory system (chapter IX)		507 (18%)	213 (194–231)	486 (18%)	219 (200–239)	21 (12·3%)	124 (71–177)
Diseases of the respiratory system (chapter X)		83 (3%)	35 (27–42)	81 (3%)	37 (29–45)	2 (1%)	12 (0–28)
Diseases of the digestive system (chapter XI)		222 (8%)	93 (81–105)	203 (8%)	92 (79–104)	19 (11%)	112 (62–163)
Symptoms, signs and abnormal clinical and laboratory findings, not elsewhere classified (chapter XVIII)		110 (4%)	46 (38–55)	106 (4%)	48 (39–57)	4 (2%)	24 (0–47)
Other non-external causes*		18 (1%)	8 (4–11)	14 (1%)	6 (3–10)	4 (2%)	24 (0–47)
External causes of morbidity and mortality (chapter XX)		1276 (44%)†	535 (506–564)	1202 (44%)†	543 (512–573)	74 (43%)	437 (337–537)
	Traffic accidents	158 (6%)	66 (56–77)	152 (6%)	69 (58–80)	6 (4%)	35 (7–64)
	Non-traffic accidents	557 (19%)	234 (214–253)	528 (20%)	238 (218–259)	29 (17%)	171 (109–234)
	Suicide	471 (16%)	198 (180–215)	435 (16%)	196 (178–215)	36 (21%)	213 (143–282)
	Homicide	83 (3%)	35 (27–42)	80 (3%)	36 (28–44)	3 (2%)	18 (0–38)

Data are n (%) or mortality per 100 000 person-years (95% CI). Causes are classified by ICD-10 chapters.

* ICD-10 chapters III, VII, VIII, XII, XIII, XIV, XV, XVI, and XVII.

† Seven deaths were categorised as ICD-10 Y35–Y98.

**Figure 1:**
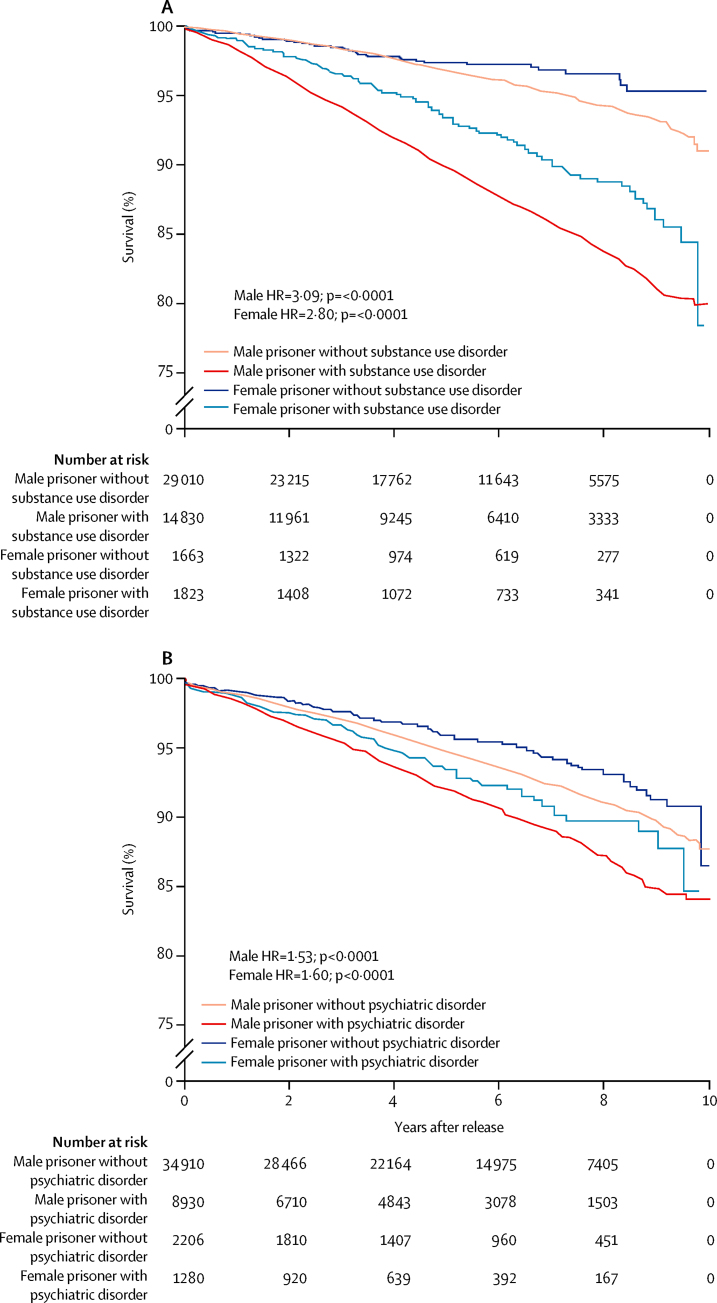
Kaplan-Meier Curves for all-cause mortality Individuals released from prison with and without (A) substance use disorders or (B) other psychiatric disorders.

The appendix shows the overall Kaplan-Meier curve for all-cause mortality in people released from prison. Individuals with previous diagnoses of substance use disorders were more likely to die than were individuals without substance use disorders (figure 1). In men, the estimated probability of death within 5 years after release was 10·2% (95% CI 9·6–10·7) for those with substance use disorders and 3·2% (3·0–3·4%) for those without. In women, the probability of death within 5 years after release was 6·5% (5·2–7·8) for those with substance use disorders and 2·6% (1·8–3·5) for those without. We identified similar differences in mortality rates in people with and without any other psychiatric disorder (figure 1).

Overall, the strongest associations were for alcohol and drug use disorders, and the associations remained after we adjusted for possible confounders (table 3). The associations between other psychiatric disorders and all-cause mortality were significant in model 1, which we adjusted for age, sex, and immigration status, but mostly disappeared after adjustment for possible confounders and alcohol and drug use disorders (models 2 and 3). We identified a similar pattern of results for the associations between individual psychiatric disorders and external-cause mortality (table 3). Sensitivity analysis for all-cause and external-cause mortality, stratified by sex, also showed a similar pattern of results (appendix). We identified similar results in analysis with multiple imputations for missing values.

**Table 3:** Association between individual psychiatric disorders and mortality after prison release

	Model 1*	**Model 2**†	**Model 3**‡
**All-cause mortality**			
Alcohol use disorder	2·11 (1·95–2·29)	1·85 (1·69–2·02)	1·62 (1·48–1·77)
Drug use disorder	2·25 (2·08–2·43)	1·90 (1·74–2·08)	1·67 (1·53–1·83)
Personality disorder	1·41 (1·24–1·61)	1·16 (1·00–1·34)	0·84 (0·72–0·98)
Attention deficit hyperactivity disorder	1·81 (1·18–2·79)	1·37 (0·83–2·24)	1·12 (0·68–1·84)
Other developmental or childhood disorder	1·41 (1·08–1·85)	1·30 (0·98–1·73)	1·17 (0·88–1·56)
Schizophrenia-spectrum disorder	1·36 (1·12–1·64)	1·13 (0·92–1·40)	0·84 (0·68–1·04)
Bipolar disorder	1·41 (0·87–2·27)	1·18 (0·68–2·04)	0·94 (0·54–1·62)
Depression	1·31 (1·14–1·51)	1·20 (1·03–1·40)	0·99 (0·85–1·16)
Anxiety disorder	1·16 (1·01–1·33)	1·12 (0·97–1·30)	0·95 (0·82–1·10)
**External-cause mortality**			
Alcohol use disorder	2·23 (1·97–2·53)	1·99 (1·73–2·28)	1·54 (1·34–1·78)
Drug use disorder	3·20 (2·85–3·59)	2·70 (2·37–3·07)	2·43 (2·13–2·78)
Personality disorder	1·92 (1·60–2·31)	1·53 (1·24–1·87)	1·01 (0·82–1·25)
Attention deficit hyperactivity disorder	1·74 (1·04–2·91)	1·53 (0·88–2·65)	1·17 (0·67–2·03)
Other developmental or childhood disorder	1·38 (1·00–1·91)	1·23 (0·87–1·74)	1·08 (0·76–1·52)
Schizophrenia-spectrum disorder	1·77 (1·36–2·32)	1·43 (1·06–1·92)	0·93 (0·69–1·26)
Bipolar disorder	1·44 (0·65–3·22)	1·09 (0·41–2·92)	0·84 (0·31–2·24)
Depression	1·74 (1·41–2·14)	1·60 (1·27–2·01)	1·24 (0·99–1·57)
Anxiety disorder	1·19 (0·97–1·46)	1·11 (0·89–1·39)	0·89 (0·71–1·11)

Data are hazard ratios (95% CI).

* Adjusted for age, sex, and immigration status.

† In addition to covariates in model 1, adjusted for sociodemographic and criminological covariates.

‡ In addition to covariates in model 2, adjusted for alcohol and drug use disorder.

**Figure 2:**
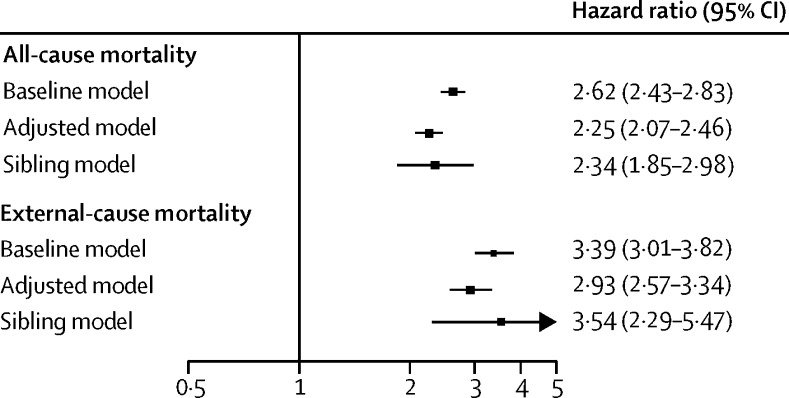
Association between substance use disorder and mortality after prison release Baseline model adjusted for age, sex, and immigration status. Adjusted model adjusted for age, sex, immigration status, and sociodemographic and criminological covariates. Sibling model adjusted for all factors shared by siblings and measured covariates, based on 1458 pairs of prisoners who were full siblings.

Because of the association between substance use disorders and post-release mortality, we further examined whether this association could be explained by familial factors by using siblings who were also imprisoned for comparison. A small proportion (20%) of siblings had overlapping sentences. The association between substance use disorders and all-cause mortality did not change substantially after adjustment for sociodemographic and criminological factors (adjusted model) and familial factors (sibling model; figure 2). The pattern of results was the same for external-cause mortality (figure 2).

Assuming causality, 2703 men died after release from prison and 925 of these deaths were potentially attributable to substance use disorders, corresponding to a PAF of 34% for all-cause mortality (appendix). In women, 85 of 171 deaths were potentially attributable to substance use (PAF 50%). For external-cause mortality, 503 of the 1202 deaths in men were potentially attributable to substance use (PAF 42%). In women, 52 of 74 external-cause deaths were potentially attributable to substance use (PAF 70%).

We examined the predictive value of substance use disorders and any other psychiatric disorders (appendix). The addition of substance use disorders to a baseline model, which included sociodemographic factors, criminological factors, and self-harm, best predicted external-cause death because it was associated with the largest increase in Harrell's c-index (5·0%; appendix). The addition of substance use disorders also significantly improved calibration and reclassification, as suggested by the likelihood ratio test (Δχ^2^ 224·4, p<0·0001) and Royston's *R*^2^(Δ% 8·1, 95% CI 2·2–13·9). Addition of any other psychiatric disorders did not further improve predictive accuracy (see appendix for HRs for all risk factors in this model). We identified the same pattern of results in relation to predictive models for all-cause mortality (data not shown).

## Discussion

In this longitudinal cohort study, substance use disorders were associated with a substantially increased rate of mortality in people released from prison, and the association remained even when we compared siblings without substance use disorders who were also in prison, which adjusts for unmeasured genetic and environmental factors. A large proportion of deaths among released prisoners could be attributed to substance use. Additionally, we identified little evidence that other psychiatric disorders increased the all-cause or external-cause mortality rate. To our knowledge, this is the first time the association between psychiatric disorders and post-release mortality has been examined with adjustment for sociodemographic, criminological, and familial factors. Our estimates of the population effect of substance use disorders on post-release mortality are also novel.

Substance use disorders were associated with a substantially increased rate of post-release mortality. More importantly, our results suggested that the association was independent from the contribution of sociodemographic, criminological, and familial factors, supporting a causal effect of substance use disorders. For individuals with substance use disorders in other settings, transitions, such as hospital discharge and discontinuation of treatment, have been associated with increased mortality.^31^, ^32^ Possible explanations include diminished physiological tolerance to substances after release^33^ and increased risk of fatal overdose after release.^3^, ^34^ Additionally, the physiological age of people in prison has been estimated to be roughly 10–15 years older than their chronological ages,^35^ and cellular and brain imaging studies have provided evidence that substance use is associated with accelerated biological ageing.^36^

Our study, with long follow-up, has several implications. First, our results show that substance use disorders had a persistent effect on risk of mortality after release, beyond the first few weeks or months. With the current focus on transitions in many clinical guidelines,^37^, ^38^ our findings suggest that this alone might not lead to large reductions in mortality of people released from prisons, and guidelines for the clinical care of these individuals need to be reviewed. Research has suggested the need to shift treatment for substance use disorder from an acute-care model to a chronic disease-management model.^39^ Future research is needed about how treatment in prison can be linked with community services and primary care to achieve a cost-effective service model with limited health-care resources. Second, although alcohol use disorder is as prevalent as drug use disorder and the increased mortality risks are similar, some countries do not give it the same level of funding or service provision.^40^ For example, in 2010–11 in England and Wales, nearly half of prisons had no alcohol-related services or programmes available.^41^, ^42^ Furthermore, in some countries, much of current provision only targets alcohol use disorder if it co-occurs with drug problems. Third, our results underscore the potential health benefits of alternatives to imprisonment, such as regular drug monitoring in the community, or diversion to treatment.^43^ Fourth, linkage to community services and adherence to treatment is likely to be important for people released from prison who have substance use disorders.^43^ Trial evidence has shown that targeted interventions, such as maintenance of opioid substitution treatment and participation in peer self-help organisations (eg, Alcoholics Anonymous), promote abstinence and produce benefits for individual health and the public good.^43^ Contingency management improves treatment adherence.^44^ Finally, appropriate and sustainable epidemiological systems will be needed to survey alcohol and drug use, overall and cause-specific mortality, and effectiveness of health-care services for populations of former prisoners.^45^, ^46^

In our study, excess mortality associated with substance use in prisoners was less than that reported for such disorders in the general population.^47^ The effect of substance use disorders on mortality in people released from prison might be less than that in the general population because prisoners have cumulative risk factors and a high baseline mortality. Another possible explanation for this difference is that we have more comprehensively adjusted for sociodemographic covariates than have similar studies in the general population.

We identified little evidence that other psychiatric disorders, including schizophrenia, bipolar disorder, and depression, independently increased post-release mortality. Although these disorders were linked with increased mortality in the initial analysis, the associations were not significant after we adjusted for sociodemographic and criminological factors and comorbid substance use disorders, suggesting that the excess mortality rate for the disorders was mainly explained by these factors. This finding was unexpected, and contrasts with previous research that has shown psychiatric disorders to increase mortality risk during incarceration.^48^ Possible explanations are that previous research did not adequately assess potential confounders, such as comorbid substance use disorders, or that the psychiatric determinants for death after release are different from those during incarceration. Alternatively, for some individuals, drug and alcohol use disorders could mask an underlying mental illness. In particular, comorbid diagnoses might be under-reported in individuals with alcohol dependency. Although we had a relatively large sample, we cannot rule out the possibility of small effects of other psychiatric disorders on post-release mortality.

PAFs assume a causal relation between substance use disorders and post-release mortality. In this study, we have not only adjusted for sociodemographic and criminological factors, but also accounted for unmeasured familial confounding by using comparisons with siblings who were released from prison. Although the results are consistent with a causal hypothesis, they remain based on observational data and need to be replicated with other study designs. Nevertheless, our results underscore the substantial contribution of substance use disorders to the increased mortality after prison release, and the PAF was increased further for external-cause mortality. In our sample, the 1276 external-cause deaths accounted for 2·7% of all external-cause mortality (47 179 deaths) in Sweden during the study period (2000–09),^24^ and substance use disorders in released prisoners contributed to 555 deaths (1·1% of all external-cause deaths). This contribution to external-cause mortality could be substantially increased in counties with higher incarceration rates than that of Sweden. For example, if our findings were replicated in the USA, about 9% of external-cause mortality in the US adult general population would potentially be preventable if substance use disorders were fully treated in all people released from jail and prison and assuming causality between substance use disorders and mortality (see appendix for calculation).

Risk assessment instruments are widely used by criminal justice systems to identify high-risk prisoners and estimate their risk of reoffending.^49^ However, little information exists on what predicts mortality after release from prison.^13^ Using the current sample, we report that substance use disorders significantly improved prediction of external-cause mortality in addition to the information available from routinely collected factors, including sociodemographic and criminological factors. Moreover, the predictive value of substance use disorders was stronger than that of other psychiatric disorders. Our findings suggest that substance use disorders, together with key sociodemographic and criminological factors, produced moderate levels of predictive accuracy and might help to identify high-risk groups for targeted intervention (eg, re-induction onto opioid substitution treatment).^50^ Further studies are needed to examine the validity of our finding in independent samples.

Therapies for substance use disorders, including pharmacological treatment, have been shown to be effective in reduction of drug misuse and related problems.^51^ However, evidence that any particular treatment can reduce mortality is not yet conclusive,^52^ which might be partly because mortality remains a rare outcome, even in high-risk groups, especially if follow-up is not sufficiently long. Further studies with extended follow-up are needed to investigate the ability of treatment for substance use disorders to reduce mortality. As the absolute numbers of people in prison with substance use disorders are large worldwide, improvements to their treatment might have a substantial effect.

Our study has three main limitations. First, the ascertainment of psychiatric diagnoses relied on data from patient registers. Although these registers have generally good diagnostic validity and the important advantage of not requiring accurate respondent recall and reporting, the prevalence of some psychiatric disorders might be underestimated (eg, ADHD and other developmental or childhood disorders). Second, the analyses did not account for the potentially ameliorative effects of treatment. Individuals who received treatment for substance use disorders would also possibly have reduced risk of adverse outcomes such as death. Consequently, our findings might be conservative estimates of the actual effect of substance use disorders on post-release mortality. Third, the findings were based on Swedish population data, and generalisation to other countries with different criminal justice systems need clarification. Although Sweden has a low incarceration rate compared with other countries,^53^ the prevalence of substance use disorders and severe psychiatric disorders reported in this study was largely similar to those in the USA and other high-income countries. For example, prevalence is estimated to be 26% for alcohol use disorder and 25% for drug use disorder in men in prison in the USA,^15^ compared with 21% for alcohol use disorder and 22% for drug use disorder in the current sample. For US female prisoners, prevalence is estimated to be 20% for alcohol use disorder and 45% for drug use disorder,^15^ compared with 28% for alcohol use disorder and 41% for drug use disorder in our sample. Furthermore, in a recent systematic review,^16^ the pooled prevalence of psychotic disorders was 3·6% in men and 3·9% in women prisoners compared with 2·8% in men and 3·7% in women in our sample.

Our results show high mortality in people released from prison, and the substantial contribution of substance use disorders to this mortality. We provide evidence that supports a causal role for substance use disorders in post-release mortality, and that these disorders might be useful to identify high-risk groups. Future research is needed to examine the cost-effectiveness of treatment for substance use disorders in custody, and to identify the best service models to maintain treatment in the community. In many countries, jails and prisons are an important opportunity to treat substance use disorders in individuals who are out of the reach of conventional health-care systems.^13^ Such efforts could not only reduce mortality in people released from custody, but also improve both public health and safety.^54^, ^55^

## Supplementary Material

Supplementary appendix

Supplementary audio Seena Fazel of Oxford University discusses how research into the health of prison populations might benefit both prisoners and society as a whole.
