# Supplementary material for: Substance use disorders, psychiatric disorders, and mortality after release from prison: a nationwide longitudinal cohort study
Source: Lancet Psychiatry. 2015 Apr 28;2(5):422–30. doi: 10.1016/S2215-0366(15)00088-7 (PMC4579558; doi:10.1016/S2215-0366(15)00088-7)
Supplement: Supplementary appendix [file mmc1.pdf]

THE LANCET Psychiatry

Supplementary appendix

This appendix formed part of the original submission and has been peer reviewed. We post it as supplied by the authors.

Supplement to: Chang Z, Lichtenstein P, Larsson H, Fazel S. Substance use disorders, psychiatric disorders, and mortality after release from prison: a nationwide longitudinal cohort study. *Lancet Psychiatry* 2015; published online April 22. http://dx.doi.org/10.1016/S2215-0366(15)00088-7.

Supplementary Table 1. Kaplan–Meier Curves for all-cause mortality in released prisoners in Sweden.

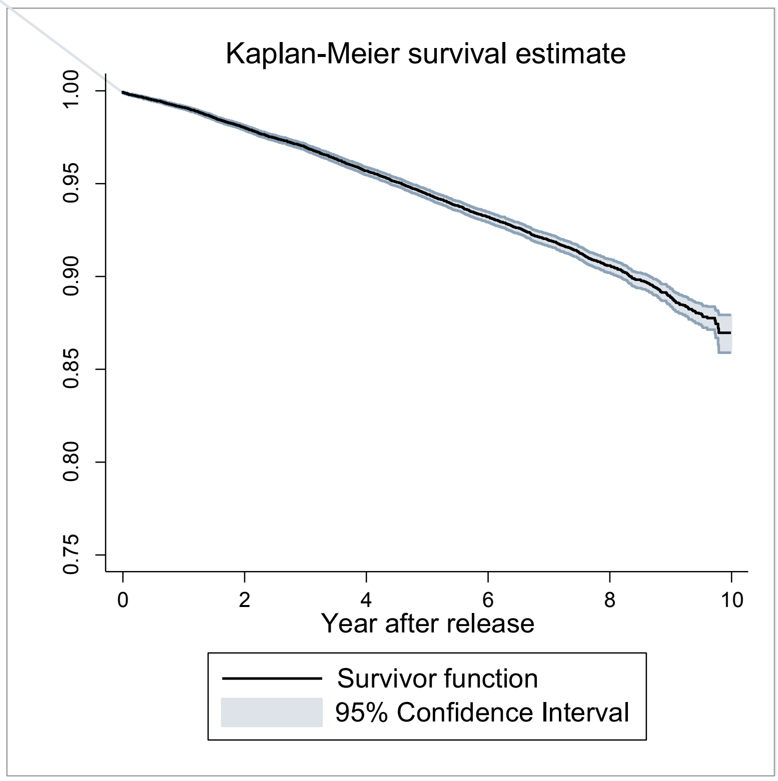

Supplementary Table 1. Association between individual diagnoses of psychiatric disorders and all-cause mortality after prison release (Hazard ratios with 95% confidence intervals).

	Model 1 [a]	Model 2 [b]	Model 3 [c]
**Male**			
Alcohol abuse	2·11 (1·95-2·29)	1·85 (1·69-2·02)	1·62 (1·48-1·77)
Drug abuse	2·25 (2·08-2·43)	1·90 (1·74-2·08)	1·67 (1·53-1·83)
Personality disorder	1·41 (1·24-1·61)	1·16 (1·00-1·34)	0·84 (0·72-0·98)
ADHD	1·81 (1·18-2·79)	1·37 (0·83-2·24)	1·12 (0·68-1·84)
Other developmental or childhood disorder	1·41 (1·08-1·85)	1·30 (0·98-1·73)	1·17 (0·88-1·56)
Schizophrenia spectrum disorder	1·36 (1·12-1·64)	1·13 (0·92-1·40)	0·84 (0·68-1·04)
Bipolar disorder	1·41 (0·87-2·27)	1·18 (0·68-2·04)	0·94 (0·54-1·62)
Depression	1·31 (1·14-1·51)	1·20 (1·03-1·40)	0·99 (0·85-1·16)
Anxiety disorder	1·16 (1·01-1·33)	1·12 (0·97-1·30)	0·95 (0·82-1·10)
**Female**			
Alcohol abuse	2·34 (1·72-3·17)	2·29 (1·64-3·20)	1·94 (1·37-2·74)
Drug abuse	2·20 (1·60-3·03)	2·33 (1·63-3·33)	1·96 (1·36-2·84)
Personality disorder	1·69 (1·09-2·61)	1·63 (1·02-2·61)	1·17 (0·72-1·89)
ADHD	1·65 (0·41-6·70)	0·93 (0·13-6·68)	0·73 (0·10-5·24)
Other developmental or childhood disorder	1·73 (0·88-3·42)	1·99 (0·99-3·99)	1·94 (0·97-3·91)
Schizophrenia spectrum disorder	2·29 (1·27-4·12)	1·87 (0·97-3·60)	1·29 (0·67-2·51)
Bipolar disorder	0·68 (0·10-4·87)	N/A	N/A
Depression	1·21 (0·77-1·90)	1·15 (0·71-1·85)	0·91 (0·56-1·48)
Anxiety disorder	1·28 (0·86-1·91)	1·16 (0·75-1·79)	1·03 (0·66-1·59)

[a] Adjusted for age and immigration status.

[b] In addition to Model 1, adjusted for socio-demographic and criminological covariates.

[c] In addition to Model 2, adjusted for alcohol and drug abuse.

Supplementary Table 2. Association between individual diagnoses of psychiatric disorders and external-cause mortality after prison release (Hazard ratios with 95% confidence intervals).

	Model 1 [a]	Model 2 [b]	Model 3 [c]
**Male**			
Alcohol abuse	2·23 (1·97-2·53)	1·99 (1·73-2·28)	1·54 (1·34-1·78)
Drug abuse	3·20 (2·85-3·59)	2·70 (2·37-3·07)	2·43 (2·13-2·78)
Personality disorder	1·92 (1·60-2·31)	1·53 (1·24-1·87)	1·01 (0·82-1·25)
ADHD	1·74 (1·04-2·91)	1·53 (0·88-2·65)	1·17 (0·67-2·03)
Other developmental or childhood disorder	1·38 (1·00-1·91)	1·23 (0·87-1·74)	1·08 (0·76-1·52)
Schizophrenia spectrum disorder	1·77 (1·36-2·32)	1·43 (1·06-1·92)	0·93 (0·69-1·26)
Bipolar disorder	1·44 (0·65-3·22)	1·09 (0·41-2·92)	0·84 (0·31-2·24)
Depression	1·74 (1·41-2·14)	1·60 (1·27-2·01)	1·24 (0·99-1·57)
Anxiety disorder	1·19 (0·97-1·46)	1·11 (0·89-1·39)	0·89 (0·71-1·11)
**Female**			
Alcohol abuse	2·09 (1·31-3·35)	2·37 (1·41-3·99)	1·76 (1·03-3·00)
Drug abuse	3·27 (1·95-5·49)	3·77 (2·08-6·84)	3·25 (1·76-5·99)
Personality disorder	2·23 (1·25-4·01)	2·34 (1·21-4·50)	1·56 (0·79-3·05)
ADHD	3·45 (0·84-14·2)	2·26 (0·31-16·5)	1·89 (0·26-13·9)
Other developmental or childhood disorder	2·06 (0·88-4·82)	2·99 (1·23-7·23)	2·64 (1·08-6·45)
Schizophrenia spectrum disorder	3·03 (1·39-6·60)	2·36 (0·93-6·00)	1·45 (0·57-3·74)
Bipolar disorder	N/A	N/A	N/A
Depression	1·94 (1·06-3·53)	2·13 (1·12-4·06)	1·67 (0·87-3·21)
Anxiety disorder	1·46 (0·81-2·61)	1·13 (0·57-2·24)	0·93 (0·47-1·86)

[a] Adjusted for age and immigration status.

[b] In addition to Model 1, adjusted for socio-demographic and criminological covariates.

[c] In addition to Model 2, adjusted for alcohol and drug abuse.

Supplementary Table 3. Population attributable fraction of substance abuse on all-cause and external-cause mortality among released prisoners.

	Total number of death	Number of individual died with substance abuse	Adjusted hazard ratio (95% CI)	Number of death attributable to substance abuse	Population attributable fraction (%, 95% CI)
All-cause mortality					
Male	2703	1689	2·22 (2·04-2·43)	925	34·2 (30·8-37·7)
Female	171	140	2·88 (1·94-4·29)	85	49·6 (33·7-63·2)
External-cause mortality					
Male	1202	826	2·86 (2·50-3·27)	503	41·8 (36·9-46·5)
Female	74	67	5·95 (2·78-12·7)	52	69·7 (48·3-84·9)

Supplementary Table 4. Incremental validity of psychiatric disorders in predicting external-cause death in released prisoners.

Model	Harrell's c-index % (95% CI)	Compare to	Δ%
I. Baseline model	65·1 (63·5 - 66·7)		
II. Baseline model + substance abuse	70·1 (68·5 - 71·6)	Model I	+5·0
III. Baseline model + any other psychiatric disorder	65·6 (63·9 – 67·2)	Model I	+0·5
IV. Baseline model + substance abuse + any other psychiatric disorder	70·1 (68·5 - 71·6)	Model II	+0·0

Note: Baseline model included age, sex, immigration status, socio-demographic factors (civil status, employment, and highest education, disposable income and neighbourhood deprivation), criminological factors (length of incarceration, violent index offence, any previous violent crime), and any previous self-harm.

Supplementary Table 5. Association between risk factors and external-cause mortality after prison release.

Risk factors	Hazard ratio (95% CI)
Substance abuse	
No	1·00 (reference)
Any	2·75 (2·40-3·15)
Sex	
Male	1·00 (reference)
Female	0·60 (0·46-0·79)
Age	0·99 (0·98-1·00)
Immigrant	0·67 (0·57-0·79)
Civil status	
Unmarried	1·00 (reference)
Married	0·89 (0·70-1·12)
Divorced	1·01 (0·86-1·19)
Widowed	1·64 (0·95-3·09)
Highest education	
< 9 yr	1·00 (reference)
9-11 yr	0·92 (0·81-1·04)
>= 12yr	1·19 (0·88-1·62)
Employed	0·53 (0·42-0·66)
Disposable income	1·00 (0·99-1·00)
Neighbourhood Deprivation	1·01 (0·96-1·06)
Length of incarceration	
< 6 mo	1·00 (reference)
6-11 mo	0·81 (0·67-0·97)
12-23 mo	0·82 (0·63-1·06)
>=24 mo	1·09 (0·75-1·62)
Violent index offence	0·90 (0·79-1·02)
Previous violent crime	1·19 (1·04-1·36)
Previous self-harm	1·39 (1·21-1·61)

Supplementary Text.

How much of the external-cause mortality in the adult general population could be prevented if substance abuse is eliminated among all ex-prisoners?

$$\% \ = \frac{[number\ of\ deaths\ in\ ex\ prisoners\ attributable\ to\ substance\ abuse]}{[number\ of\ deaths\ in\ thegeneral\ population\ ]} \quad (1)$$

$$\% \ = \frac{[ex\ prisoner\ populaiton]*[mortality\ rate\ in\ ex\ priosners]*[PAF]}{[general\ population]*[mortality\ rate\ in\ general\ population]} \quad (2)$$

$$\% \ = [propotion\ of\ ex\ prisoenrs\ in\ population] * [mortatlity\ ratio] * [PAF] \quad (3)$$

PAF = Population Attributable Fraction.

In the current sample, the 1276 external-cause deaths accounted for 2.7% of all external-cause deaths (47179) in Sweden during the study period (2000-2009).[1] Using the PAF of 43.5%, about 1.2% of external-cause mortality in the adult general population could be prevented if substance abuse is eliminated among all ex-prisoners.

In the US, ex-prisoners are estimated to account for 3·3% of the US adult population,[1] and the mortality ratio for external-cause mortality is 6·5.[2] From our results, we estimated the PAF on external-cause mortality from substance abuse is 43·5%. Using (3), and if our estimate of PAF is generalizable, we have: $\% \ = 3.3\% * 6.5 * 43.5\%$ = 9.3%

1. Cause of Death Register. The Swedish National Board of Health and Welfare (Socialstyrelsen). Accessed Jan 2015. http://www.socialstyrelsen.se/statistik/statistikdatabas/dodsorsaker

2. Shannon S, Uggen C, Thompson M, Schnittker J, Massoglia M. Growth in the U.S. Ex-Felon and Ex-Prisoner Population, 1948 to 2010. Annual Meetings of the Population Association of America 2011.

3. Binswanger IA, Stern MF, Deyo RA, et al. Release from prison--a high risk of death for former inmates. *The New England journal of medicine* 2007; **356**(2): 157-65.

STROBE Statement—checklist of items that should be included in reports of observational studies

	Item No	Recommendation	Page #
**Title and abstract**	1	(*a*) Indicate the study's design with a commonly used term in the title or the abstract	1
		(*b*) Provide in the abstract an informative and balanced summary of what was done and what was found	2
**Introduction**			
Background/rationale	2	Explain the scientific background and rationale for the investigation being reported	3
Objectives	3	State specific objectives, including any prespecified hypotheses	3
**Methods**			
Study design	4	Present key elements of study design early in the paper	4
Setting	5	Describe the setting, locations, and relevant dates, including periods of recruitment, exposure, follow-up, and data collection	4
Participants	6	(*a*) *Cohort study*—Give the eligibility criteria, and the sources and methods of selection of participants. Describe methods of follow-up<br>*Case-control study*—Give the eligibility criteria, and the sources and methods of case ascertainment and control selection. Give the rationale for the choice of cases and controls<br>*Cross-sectional study*—Give the eligibility criteria, and the sources and methods of selection of participants	4
		(*b*) *Cohort study*—For matched studies, give matching criteria and number of exposed and unexposed<br>*Case-control study*—For matched studies, give matching criteria and the number of controls per case	n/a
Variables	7	Clearly define all outcomes, exposures, predictors, potential confounders, and effect modifiers. Give diagnostic criteria, if applicable	4-5
Data sources/ measurement	8*	For each variable of interest, give sources of data and details of methods of assessment (measurement). Describe comparability of assessment methods if there is more than one group	4-5
Bias	9	Describe any efforts to address potential sources of bias	6
Study size	10	Explain how the study size was arrived at	4
Quantitative variables	11	Explain how quantitative variables were handled in the analyses. If applicable, describe which groupings were chosen and why	5
Statistical methods	12	(*a*) Describe all statistical methods, including those used to control for confounding	5-6
		(*b*) Describe any methods used to examine subgroups and interactions	8
		(*c*) Explain how missing data were addressed	5
		(*d*) *Cohort study*—If applicable, explain how loss to follow-up was addressed<br>*Case-control study*—If applicable, explain how matching of cases and controls was addressed<br>*Cross-sectional study*—If applicable, describe analytical methods taking account of sampling strategy	4
		(*e*) Describe any sensitivity analyses	8

**Results**			
Participants	13*	(a) Report numbers of individuals at each stage of study—eg numbers potentially eligible, examined for eligibility, confirmed eligible, included in the study, completing follow-up, and analysed	4
		(b) Give reasons for non-participation at each stage	--
		(c) Consider use of a flow diagram	--
Descriptive data	14*	(a) Give characteristics of study participants (eg demographic, clinical, social) and information on exposures and potential confounders	7, 21
		(b) Indicate number of participants with missing data for each variable of interest	21
		(c) *Cohort study*—Summarise follow-up time (eg, average and total amount)	7, 21
Outcome data	15*	*Cohort study*—Report numbers of outcome events or summary measures over time	7
		Case-control study—Report numbers in each exposure category, or summary measures of exposure	
		Cross-sectional study—Report numbers of outcome events or summary measures	
Main results	16	(*a*) Give unadjusted estimates and, if applicable, confounder-adjusted estimates and their precision (eg, 95% confidence interval). Make clear which confounders were adjusted for and why they were included	20
		(*b*) Report category boundaries when continuous variables were categorized	n/a
		(*c*) If relevant, consider translating estimates of relative risk into absolute risk for a meaningful time period	n/a
Other analyses	17	Report other analyses done—eg analyses of subgroups and interactions, and sensitivity analyses	8
**Discussion**			
Key results	18	Summarise key results with reference to study objectives	9
Limitations	19	Discuss limitations of the study, taking into account sources of potential bias or imprecision. Discuss both direction and magnitude of any potential bias	11-12
Interpretation	20	Give a cautious overall interpretation of results considering objectives, limitations, multiplicity of analyses, results from similar studies, and other relevant evidence	12
Generalisability	21	Discuss the generalisability (external validity) of the study results	11-12
**Other information**			
Funding	22	Give the source of funding and the role of the funders for the present study and, if applicable, for the original study on which the present article is based	7

*Give information separately for cases and controls in case-control studies and, if applicable, for exposed and unexposed groups in cohort and cross-sectional studies.

Note: An Explanation and Elaboration article discusses each checklist item and gives methodological background and published examples of transparent reporting. The STROBE checklist is best used in conjunction with this article (freely available on the Web sites of PLoS Medicine at http://www.plosmedicine.org/, Annals of Internal Medicine at http://www.annals.org/, and Epidemiology at http://www.epidem.com/). Information on the STROBE Initiative is available at www.strobe-statement.org
